# Der Schwangerschaftsabbruch auf YouTube, Instagram und TikTok: Eine Inhalts- und Qualitätsanalyse

**DOI:** 10.1007/s00103-025-04170-x

**Published:** 2025-12-12

**Authors:** Nicola Döring, Anastasiia Shevtsova, Claudia Schumann-Doermer

**Affiliations:** 1 Institut für Medien und Kommunikationswissenschaft (IfMK), Technische Universität Ilmenau, Ehrenbergstraße 29, 98693 Ilmenau, Deutschland; 2 Deutsche Gesellschaft für psychosomatische Frauenheilkunde und Geburtshilfe (DGPFG), Dresden, Deutschland

**Keywords:** Abtreibung, Gesundheitsinformationen, mDISCERN-Index, Schwangerschaftsabbruch, Soziale Medien, Abortion, Health information, mDISCERN index, Pregnancy termination, Social media

## Abstract

**Hintergrund:**

Jugendliche und Erwachsene beziehen Informationen über den Schwangerschaftsabbruch (SAB) zunehmend über soziale Medien. Vor diesem Hintergrund ist es Ziel der vorliegenden Studie, erstmals Inhalte und Qualität deutschsprachiger SAB-Videos auf YouTube, Instagram und TikTok zu untersuchen. Beantwortet werden sollen Forschungsfragen zu Anbietertypen (Forschungsfrage 1: F1), Inhalten (F2) und Qualität der SAB-Videos (F3) sowie zu Publikumsreaktionen (F4).

**Methoden:**

Es wurde eine Stichprobe von *N* = 500 populären SAB-Videos von YouTube (150), Instagram (150) und TikTok (200) gezogen. Pro Video gingen die maximal 20 meistgelikten themenbezogenen Kommentare in das Sample ein (*N* = 4761). Die Videos und Kommentare wurden mittels reliabilitätsgeprüfter Codebücher analysiert. Die Datenanalyse erfolgte mit R. Die Studie ist präregistriert und alle Daten, Materialien und Analyseskripte sind öffentlich verfügbar.

**Ergebnisse:**

Die SAB-Videos stammten überwiegend von Medienprofis (49 %) und nur selten von Gesundheitsprofis (6 %; F1). Inhaltlich vertraten die SAB-Videos mehrheitlich eine Pro-Choice-Position (54 %) und sprachen häufig die medizinische Versorgung sowie psychisches und physisches Erleben an (F2). Gemäß Qualitätskriterien für Gesundheitsinformationen zeigten sich deutliche Defizite, wobei YouTube-Videos am besten abschnitten (F3). TikTok-Videos dagegen waren führend bei Publikumsreaktionen wie Views, Likes und Kommentaren. Die Kommentarspalten wurden vom Publikum genutzt, um sich politisch zu positionieren und um persönliche Erfahrungen zu teilen (F4).

**Diskussion:**

Weitere Forschung sowie Praxismaßnahmen sind notwendig, um die Qualität von Social-Media-Videos zum Schwangerschaftsabbruch besser einschätzen und optimieren zu können.

## Abstract (en)

**Background:**

Young people and adults increasingly obtain information about pregnancy termination (PT) through social media. Against this background, the aim of this study is to investigate, for the first time, the content and quality of German-language PT videos on YouTube, Instagram, and TikTok. Research questions on provider types (research question 1: RQ1), content (RQ2), and quality of PT videos (RQ3) as well as audience reactions (RQ4) will be answered.

**Methods:**

A sample of *N* = 500 popular PT videos was drawn from YouTube (150), Instagram (150), and TikTok (200). For each video, up to 20 of the most liked topic-related audience comments were included in the sample (*N* = 4761). The videos and comments were analyzed using reliability-tested codebooks. Data analysis was conducted with R. The study is preregistered, and all data, materials, and analysis scripts are publicly available.

**Results:**

The PT videos predominantly originated from media professionals (49%) and only rarely from healthcare professionals (6%; RQ1). In terms of content, the majority of the videos represented a pro-choice position (54%) and frequently addressed medical care as well as psychological and physical experience (RQ2). According to quality criteria for health information, clear deficits were identified, with YouTube videos performing best in comparison (RQ3). TikTok videos, by contrast, led in audience engagement, recording the highest numbers of views, likes, and comments. Viewers used comment sections to express political positions and share personal experiences (RQ4).

**Discussion:**

Future research as well as practice interventions are needed to further assess the quality of social media videos on pregnancy termination and improve it in a more targeted way.

## Hintergrund

Unter einem *Schwangerschaftsabbruch* (kurz: SAB; auch: Abbruch, Abtreibung) versteht man das durch äußere Einwirkung herbeigeführte vorzeitige Beenden einer Schwangerschaft [1, 2]. Unmittelbar betroffen von Schwangerschaft und möglichem Schwangerschaftsabbruch sind Mädchen und Frauen im gebärfähigen Alter (Kerngruppe: 15 bis 45 Jahre). Inklusiver ist kontextspezifisch von gebärfähigen bzw. schwangeren Personen zu sprechen, da z. B. auch trans Männer Kinder bekommen können [3].

In der internationalen Gesundheitsforschung, Versorgungspolitik und im Völkerrecht wird das Recht auf reproduktive Autonomie – also die selbstbestimmte Entscheidung über Fruchtbarkeit, Fortpflanzung und Familienplanung – heute als zentrales sexuelles und reproduktives Menschenrecht anerkannt [4–7]. Voraussetzung für die Umsetzung dieses Rechts ist ein niedrigschwelliger Zugang zu evidenzbasierten Informationen und medizinischen Versorgungsangeboten bezüglich (a) Methoden der Schwangerschaftsverhütung sowie (b) Methoden des Schwangerschaftsabbruchs (z. B. bei Versagen von Verhütungsmethoden). Weltweit endet etwa jede dritte Schwangerschaft (30 %) in einem Schwangerschaftsabbruch [7].

In Deutschland ist der Schwangerschaftsabbruch laut § 218 des Strafgesetzbuchs (StGB) illegalisiert, aber gemäß § 218a Abs. 1 StGB (Fristenregelung) in den ersten 3 Monaten straffrei gestellt, sofern er auf Wunsch der Schwangeren von ärztlichem Fachpersonal durchgeführt wird und mindestens 3 Tage zuvor eine Schwangerschaftskonfliktberatung in Anspruch genommen wurde.^1^ In der 20. Legislaturperiode des Deutschen Bundestages wurde die rechtliche Regelung des Schwangerschaftsabbruchs gemäß Koalitionsvertrag auf den Prüfstand gestellt. Die dafür eingesetzte Kommission für reproduktive Selbstbestimmung und Fortpflanzungsmedizin (KOMrSF) empfiehlt unter anderem, den Abbruch einer Frühschwangerschaft (erste 3 Monate) zukünftig außerhalb des Strafgesetzbuches zu regeln [8]. Ob und wann die Empfehlungen der Kommission umgesetzt werden, ist aktuell unklar. Zu beachten ist aus Sicht der öffentlichen Gesundheit, dass die bisherige Kriminalisierung des Schwangerschaftsabbruchs in Deutschland durch Regelung im Strafgesetzbuch mit einer starken Stigmatisierung sowohl von Ärzt*innen als auch Patient*innen einhergeht, die Abbrüche anbieten bzw. nachfragen [9].

In Deutschland werden relativ konstant jährlich rund 100.000 Schwangerschaftsabbrüche vorgenommen [10]. Rund jede vierte Frau in Deutschland lässt laut der vom Bundesinstitut für Öffentliche Gesundheit (BIÖG) geförderten repräsentativen Studie „frauen leben 4“ im Laufe ihres Lebens mindestens einen Schwangerschaftsabbruch durchführen [11, 12]. Aus dieser recht hohen Verbreitung leiten sich ein großer Versorgungs- und Informationsbedarf ab.

Hinsichtlich Versorgung zeigen sich in Deutschland jedoch deutliche Defizite, sodass ungewollt Schwangere oft Probleme haben, wohnort- und zeitnah Beratungs- und Behandlungstermine zu bekommen [9]. Hinsichtlich Informationslage werden ebenfalls Mängel beklagt. So führt die Stigmatisierung des Schwangerschaftsabbruchs dazu, dass Betroffene sich ungern offenbaren [13] und sich nicht selten gezwungen sehen, den Abbruch vor ihrem sozialen Umfeld zu verheimlichen, was ihnen Informations- und Unterstützungsressourcen entzieht [9]. In der schulischen Sexualaufklärung wird der Schwangerschaftsabbruch eher selten behandelt [14]. Aktuelle Ratgeberbücher wollen die Rolle einer „guten Freundin“ übernehmen und Unterstützung und Informationen liefern (z. B. [15]). Letztlich ist es aber doch das Internet, das für große Bevölkerungskreise zu einer zentralen Informationsquelle und Kommunikationsplattform über sexuelle und reproduktive Gesundheit im Allgemeinen [16–18] sowie auch über den Schwangerschaftsabbruch im Besonderen [19, 20] geworden ist.

Wer heute nach „Schwangerschaftsabbruch“ oder „Abtreibung“ googelt, findet auf den oberen Plätzen der Trefferliste häufig eine mithilfe von künstlicher Intelligenz (Gemini) erstellte Zusammenfassung [21, 22], zudem Wikipedia-Einträge, Gesundheitsportale sowie Webseiten von Fachorganisationen wie pro familia und BIÖG, aber auch von Arztpraxen^2^ und Beratungsstellen zum Schwangerschaftskonflikt [23].

Viele junge Menschen starten inzwischen ihre Online-Informationssuche zum Thema SAB jedoch nicht mehr über die Suchmaschine Google, sondern über die Suchmasken von Social-Media-Plattformen wie YouTube, Instagram und TikTok. Aktuelle internationale [20, 24] sowie erste nationale Studien [19, 25, 26] dokumentieren eine Fülle an SAB-Videos in sozialen Medien.

Vor diesem Hintergrund ist es Ziel der vorliegenden Studie, erstmals systematisch und in größerem Umfang für den deutschsprachigen Raum zu untersuchen, wie und von wem der Schwangerschaftsabbruch in Informationsvideos auf den führenden Social-Media-Plattformen YouTube, Instagram und TikTok dargestellt wird und wie das Publikum online auf diese Inhalte reagiert.

### Forschungsstand.

Die bisherige internationale Medieninhaltsforschung zum Schwangerschaftsabbruch in sozialen Medien konzentriert sich auf politische und gesundheitliche Aspekte:

- Mit Blick auf *politische Kommunikation* wird betrachtet, welche Positionen zur rechtlichen Regulierung des Schwangerschaftsabbruchs in sozialen Medien vertreten werden – zwischen dem Recht auf reproduktive Autonomie, einschließlich Entscheidung für einen Abbruch, einerseits (sog. Pro-Choice-Position) und dem Lebensrecht von Fötus/Embryo und Verbot von Abtreibungen andererseits (sog. Pro-Life-Position). So zeigen Inhaltsanalysen englischsprachiger Social-Media-Beiträge teilweise eine starke Polarisierung, einschließlich Hassrede, zwischen beiden Positionen [27, 28]. Studien zeichnen nach, welche Botschaften die Antiabtreibungsbewegung auf Instagram verbreitet [29] oder wie sie Pro-Choice-Hashtags kapert [30]. Ebenso wird untersucht, wie präsent Pro-Choice-Positionen auf X/Twitter [31], YouTube [27], Instagram [32] und TikTok [33, 34] sind und wie sie begründet werden.
- Mit Blick auf *Gesundheitskommunikation* wird untersucht, inwiefern in sozialen Medien sachgerechte Informationen zu psychosozialen, gesundheitlichen und medizinischen Aspekten des Schwangerschaftsabbruchs ausgetauscht werden, die Betroffenen und ihren Angehörigen helfen, eine informierte Entscheidung zu treffen und diese umzusetzen. Hier weisen Studien zu englischsprachigen Social-Media-Beiträgen immer wieder auf nennenswerte Fehlerraten hin. Fehlinformationen betreffen beispielsweise die Verbreitung von empirisch widerlegten sogenannten Abtreibungsmythen [2], denen gemäß ein Abbruch angeblich meist schwerwiegende negative Gesundheitsfolgen für die Frau hat (z. B. Depressionen, Unfruchtbarkeit). Zu den Fehlinformationen zählen aber auch angeblich wirksame „Hausmittel“ für einen Abbruch ohne medizinische Begleitung. Untersuchungen von SAB-Videos auf YouTube [35, 36] und TikTok [34, 36] zeigten anhand unterschiedlicher Stichproben und verschiedener Bewertungskriterien äußerst heterogene Ergebnisse zwischen 100 % SAB-Videos auf YouTube (von *N* = 32) mit unzureichender Informationsqualität [35] und 5 % SAB-Videos auf TikTok (von *N* = 143) mit fehlerhaften Informationen [36].

In der Gesamtschau zeigen die vorliegenden Analysen englischsprachiger Social-Media-Inhalte zum Schwangerschaftsabbruch (a) polarisierte politische Debatten, wobei Pro-Choice-Positionen oft dominieren, sowie (b) eine mangelhafte Qualität der Gesundheitsinformationen mit nennenswerten Raten an Defiziten (z. B. unvollständige oder fehlerhafte Informationen).

### Forschungsziel.

Da sich die bisherige Forschung vor allem auf englischsprachiges Material bezieht und meist nur einzelne Plattformen betrachtet, zielt die vorliegende Untersuchung darauf ab, auf der Basis eigener qualitativer [25, 26] und quantitativer Vorstudien [19, 37] erstmals einen breiten quantitativen Überblick über deutschsprachige Social-Media-Videos zum Schwangerschaftsabbruch zu geben und dabei die 3 führenden Plattformen YouTube, Instagram und TikTok vergleichend einzubeziehen. Dementsprechend sind folgende Forschungsfragen zu beantworten:

- F1: Wer bietet auf YouTube, Instagram und TikTok deutschsprachige Informationsvideos über den Schwangerschaftsabbruch an?
- F2: Welche Inhalte haben diese Informationsvideos, etwa bezüglich politischer Positionen und Gesundheitsaufklärung?
- F3: Welche Qualität haben diese Informationsvideos bezüglich Qualitätskriterien für Gesundheitsinformationen und Verbreitung von Abtreibungsmythen?
- F4: Welche Publikumsreaktionen zeigen sich bei diesen Informationsvideos (Anzahl der Views, Likes und Kommentare sowie Inhalte der Kommentare)?

## Methoden

### Untersuchungsdesign

Zur Beantwortung der 4 Forschungsfragen wurde im Jahr 2024 eine manuelle Inhalts- und Qualitätsanalyse als quantitative Querschnittstudie durchgeführt [38]. Die Studie ist präregistriert und folgt dem Open-Science-Ansatz, das heißt, alle Codebücher, Datensätze, Auswertungsskripte sowie zusätzliche Ergebnistabellen und -abbildungen Z1–Z4 sind auf dem Server des Open Science Framework (OSF) hinterlegt: https://osf.io/9ew4k/. Das Untersuchungsmaterial besteht aus öffentlich zugänglichen Social-Media-Videos und zugehörigen Kommentaren, die nach aktuellem Verständnis der Online-Forschungsethik für wissenschaftliche Untersuchungen frei zur Verfügung stehen [39].^3^ Stichprobenbildung, Instrument sowie Datenerhebung und Datenanalyse werden im Folgenden erläutert.

### Stichprobenbildung

Die Studie basiert auf einer Stichprobe von *N* = 500 Videos zum Schwangerschaftsabbruch von YouTube, Instagram und TikTok und *N* = 4761 zugehörigen Publikumskommentaren. Die SAB-Videos wurden über die Suche nach „Schwangerschaftsabbruch“ und „Abtreibung“ identifiziert. Es wurden die Top-Videos aus den jeweiligen Plattform-Rankings ausgewählt. Eingeschlossen wurden nur deutschsprachige Videos, die den Schwangerschaftsabbruch als zentrales Thema sachlich behandeln. Zu jedem Video wurden die 20 meistgelikten Top-Level-Publikumskommentare mit inhaltlichem Bezug zu SAB erhoben. Da nicht zu allen Videos 20 themenbezogene Kommentare existierten (sondern im Mittel rund 10), ergab sich ein Sample, das kleiner ist als das theoretische Maximalsample von 10.000 Kommentaren. Ausgeschlossen wurden Publikationskommentare ohne SAB-Bezug (z. B. Grüße, Witze, Werbung). Die Zusammensetzung der Stichproben ist Tab. 1 zu entnehmen. Die Entscheidung, jeweils Top-Videos und Top-Kommentare auszuwählen, basiert auf der Überlegung, dass diese Beiträge und Kommentare die höchste Wahrscheinlichkeit haben, vom Publikum wahrgenommen zu werden.

**Tab. 1:** Zusammensetzung der Stichprobe von Social-Media-Videos zum Schwangerschaftsabbruch (SAB) und zugehörigen themenbezogenen Kommentaren

	YouTube	Instagram	TikTok
Anzahl der Top-Videos (*N* = 500)	150	150	200
Suchbegriff(e)	Schwangerschaftsabbruch	Schwangerschaftsabbruch	Schwangerschaftsabbruch
	Abtreibung	Abtreibung	Abtreibung
Auswahl der Videos	„Inkognito“-Auswahl nach Reihenfolge der von YouTube präsentierten deutschsprachigen Suchergebnisse nach YouTubes Relevanz ohne Veränderung der Filtereinstellungen	Ein neuer Forschungsaccount wurde erstellt. Auswahl nach Reihenfolge der von Instagram präsentierten deutschsprachigen Suchergebnisse unter der „Für-dich“-Kategorie via Android-Emulator „BlueStacks“	Ein neuer Forschungsaccount wurde erstellt. Auswahl nach Reihenfolge der von TikTok präsentierten deutschsprachigen Suchergebnisse ohne Veränderung der Filtereinstellungen
*Video-Länge (Minuten)*			
Minimum	00:25	00:05	00:05
Maximum	29:34	16:58	07:40
Mittelwert	09:13	01:54	01:09
Median	06:06	01:00	00:54
Anzahl der Top-Kommentare (*N* = 4761)	1621	918	2222
Auswahl der Kommentare	Top 20 meistgelikte Kommentare zum Thema SAB pro Video	Top 20 meistgelikte Kommentare zum Thema SAB pro Video	Top 20 meistgelikte Kommentare zum Thema SAB pro Video
*Kommentarlänge (Wörter)*			
Minimum	1	1	1
Maximum	1457	277	222
Mittelwert	66	40	18
Median	39	26	18

Größeres Teilsample für TikTok wegen der Kürze der TikTok-Videos

### Instrument

Zur Beantwortung der Forschungsfragen wurde ein Codebuch für die Videos und eines für die Kommentare entwickelt, teils induktiv anhand des Materials, teils deduktiv anhand der Fachliteratur. Zudem wurde bei der Entwicklung und Validierung des Codebuchs auf die Expertise einer gynäkologischen Fachärztin (Autorin 3) zurückgegriffen. Das Video-Codebuch gliedert sich in folgende 5 Teile:

1. *Formale Variablen*: Sie erfassen allgemeine Merkmale der Videos (z. B. Link zum Video, Titel des Videos, Veröffentlichungsdatum, Videolänge).
2. *Typ des Video-Anbieters*: Zur Beantwortung von F1 wurden verschiedene Typen von Informationsanbietern differenziert. Besonders wichtig war hier gemäß der Fachliteratur zur Online-Gesundheitskommunikation (z. B. [16, 41]), ob SAB-Videos von Gesundheitsprofis stammen (z. B. von Gynäkolog*innen) oder von Gesundheitslaien (z. B. von Patient*innen). Weitere wichtige Anbietertypen sind Medienprofis (z. B. Journalist*innen) sowie politische und religiöse Akteure. Sofern es sich beim Informationsanbieter um eine natürliche Person handelte, wurde das Geschlecht erfasst.
3. *Video-Inhalte*: Zur Beantwortung von F2 wurde in Anlehnung an den Forschungsstand erfasst: (1) welche politische Position zum Schwangerschaftsabbruch vertreten wird (etwa die „Pro-Choice“- oder die „Pro-Life“-Position [37]) und (2) welche Gesundheitsinformationen bereitgestellt werden. Letztere wurden laut Fachliteratur in 8 Aspekte aufgeteilt, nämlich (1) medizinische Aspekte (z. B. chirurgische und medikamentöse Abbruchmethode), (2) versorgungsbezogene Aspekte (z. B. medizinische Erstberatung, Schwangerschaftskonfliktberatung), (3) erlebensbezogene Aspekte (z. B. psychisches und physisches Erleben des Abbruchs), (4) soziale und gesellschaftliche Aspekte (z. B. Stigmatisierung, Unterstützung), (5) ökonomische Aspekte (z. B. Kosten für den Abbruch), (6) rechtliche Aspekte (z. B. nationale und internationale Rechtslage), (7) ethische Aspekte (z. B. moralische Überlegungen) und (8) wissensbezogene Aspekte (z. B. Verfügbarkeit von Informationsmaterialien über den Abbruch [2, 42, 43]). Schließlich wurde in Anlehnung an frühere Studien [44] auch die Art der präsentierten Informationen (Faktenwissen und/oder Erfahrungswissen) erfasst.
4. *Inhaltsqualität* der Videos: Zur Beantwortung von F3 wurden die Videos mit dem etablierten Messinstrument zur Qualität von Online-Gesundheitsinformationen beurteilt, dem DISCERN-Index (wobei DISCERN die englische Bezeichnung für „Bewerten“ ist) [45]. In der vorliegenden Analyse wurde der modifizierte DISCERN-Index (mDISCERN) für soziale Medien verwendet [46]. Der mDISCERN-Index ist das meistgenutzte Instrument zur Messung der Qualität von Social-Media-Gesundheitsinformationen und beinhaltet 5 Qualitätskriterien: 1. die Nennung der Ziele eines Beitrags, 2. die Verwendung von zuverlässigen Informationsquellen, 3. die ausgewogene und unvoreingenommene Informationsdarstellung, 4. die Angabe von weiterführenden Informationen und 5. die Nennung von Kontroversen oder Unsicherheiten. Die Beurteilung der Einzelkriterien fließt in einen mDISCERN-Gesamtwert ein mit einem Wertebereich von 0 (schlechteste Qualität) bis 5 (beste Qualität). Weiterhin wurden aus der Fachliteratur die gängigsten Mythen über Schwangerschaftsabbrüche [2] entnommen und ihr Auftreten in den Videos wurde codiert.
5. *Quantitative Publikumsreaktionen*: Zur Beantwortung von F4 wurden für jedes Video (1) die Anzahl der Views, (2) die Anzahl der Likes und (3) die Anzahl der vorhandenen Kommentare im Erhebungszeitraum (30.04.2024–18.06.2024) erfasst. Diese Social-Media-Metriken werden unter dem jeweiligen Social-Media-Beitrag angegeben und zeigen, wie stark das Publikum mit dem Beitrag interagiert.

Das Codebuch für die Video-Kommentare gliedert sich in 2 Blöcke:

1. *Formale Variablen:* Sie erfassen, auf welches Video sich der Kommentar bezieht und dokumentieren den Wortlaut des Kommentars sowie die Kommentarlänge.
2. *Qualitative Publikumsreaktionen: *Inhalte der Kommentare wurden anhand früherer Studien [37, 44] codiert in die 4 Typen von Reaktionen: 1. Nachfrage, 2. Zusatzinformation, 3. eigene Erfahrung, 4. sowie politische Positionierung, etwa „Pro Choice“ oder „Pro Life“.

Die Reliabilität der Kategorien wurde anhand von 50 zufällig ausgewählten Videos und 300 zufällig ausgewählten Kommentaren jeweils von 2 geschulten unabhängigen Codierenden erfasst. Berechnet wurde der im Feld der Medieninhaltsforschung etablierte Reliabilitätskoeffizient Gwets AC1, der mit Mittelwerten von 0,89 für Videos und 0,94 für Kommentare auf sehr gute Messgenauigkeit hinweist ([47]; Zusatztabelle Z1 mit allen Reliabilitätskoeffizienten ist dem OSF-Projekt zu entnehmen).

### Datenerhebung und Datenanalyse

Die Datenerhebung erfolgte im Rahmen manueller Codierung durch 2 geschulte Codierende. Grundlage der Codierung waren dabei die oben dargestellten beiden reliabilitätsgeprüften Codebücher. Die Datenanalyse erfolgte deskriptiv- und inferenzstatistisch (Häufigkeitsanalysen, Chi-Quadrat-Tests bzw. Fishers exakte Tests und Varianzanalysen) unter Nutzung der Software R (R-Pakete ‚DescTools‘, ‚expss‘, ‚stats‘, ‚irrCAC‘).

## Ergebnisse

### Anbietertypen von SAB-Videos in sozialen Medien (F1)

Rund die Hälfte aller untersuchten deutschsprachigen Top-Videos zum Schwangerschaftsabbruch stammte von Medienprofis (Tab. 2): Überwiegend auf journalistische Darstellungen stößt, wer auf YouTube (71 %) oder Instagram (56 %) nach SAB-Informationen sucht und dabei die populären SAB-Videos angezeigt bekommt. Ein Beispiel ist das 15-minütige YouTube-Video „Abtreibung: Das kommt auf mich zu!“ vom Format „Puls Reportage“ des Bayerischen Rundfunks. Hier erklärt die Journalistin Ariane Schritt für Schritt, was nach einem positiven Schwangerschaftstest auf dem Weg zum Abbruch zu tun ist: Das Publikum begleitet Ariane zu einer pro-familia-Beratungsstelle, dann zu einer Frauenärztin und lernt dabei das Versorgungssystem kennen.^4^ Auch auf TikTok sind Ausschnitte aus journalistischen Reportagen präsent, allerdings deutlich seltener (27 %).

**Tab. 2:** Anbietertypen der Top-Videos zum Schwangerschaftsabbruch auf YouTube, Instagram und TikTok (in Prozent der Videos im Sample)

Anbietertyp	Gesamt	YouTube	Instagram	TikTok	*p*
	*N* = 500	*n* = 150	*n* = 150	*n* = 200	
Medienprofi	48,8	70,7	56,0	27,0	< 0,001
Gesundheitslaie	27,8	7,3	11,3	55,5	< 0,001
Politikakteur	6,6	0,7	15,3	4,5	< 0,001
Gesundheitsprofi	6,4	9,3	4,7	5,5	0,204
Religionsvertreter	5,8	8,7	6,7	3,0	0,070
Sonstiges/unklar	4,6	3,3	6,0	4,5	0,543

Prozentwerte basieren auf den Top-Videos zum Schwangerschaftsabbruch und sind nach den Werten in der Gesamtspalte absteigend sortiert. Zeilenweise Auswertung mit zweidimensionalen Chi-Quadrat-Tests. Gesamttest: zweidimensionaler Chi-Quadrat-Test (Anbietertyp × Plattform), *χ*^*2*^(10) = 164,04, *p* < 0,001, Cramérs *V* = 0,41

Auf TikTok dominieren dafür SAB-Videos von Gesundheitslaien (56 %), typischerweise Frauen, die ihre persönlichen Erfahrungen mit einem Abbruch teilen. Ein Beispiel ist das 40-sekündige TikTok-Video „Nicht jede Abtreibung ist mit Leid verbunden“ der jungen Influencerin Suki. Es beginnt mit den Worten: „Ich will heute über Abtreibungen reden, weil ich hab’ selbst mal abgetrieben. Und ich will meine Erfahrungen mit euch teilen.“ Suki erzählt, wie sie mit 19 Jahren bei einem Australienaufenthalt ungeplant schwanger wurde und dass der Abbruch für sie genau die richtige Entscheidung war.^5^ Auf Instagram (11 %) und YouTube (7 %) sind solche persönlichen Bekenntnisse unter den Top-Videos vergleichsweise selten.

Gesundheitsprofis, Politikakteure, Religionsvertreter und sonstige Personen oder Gruppen spielen als Anbietertypen von reichweitenstarken SAB-Videos eine untergeordnete Rolle (jeweils rund 6 %).

### Inhalte von SAB-Videos in sozialen Medien (F2)

**Abb. 1:**
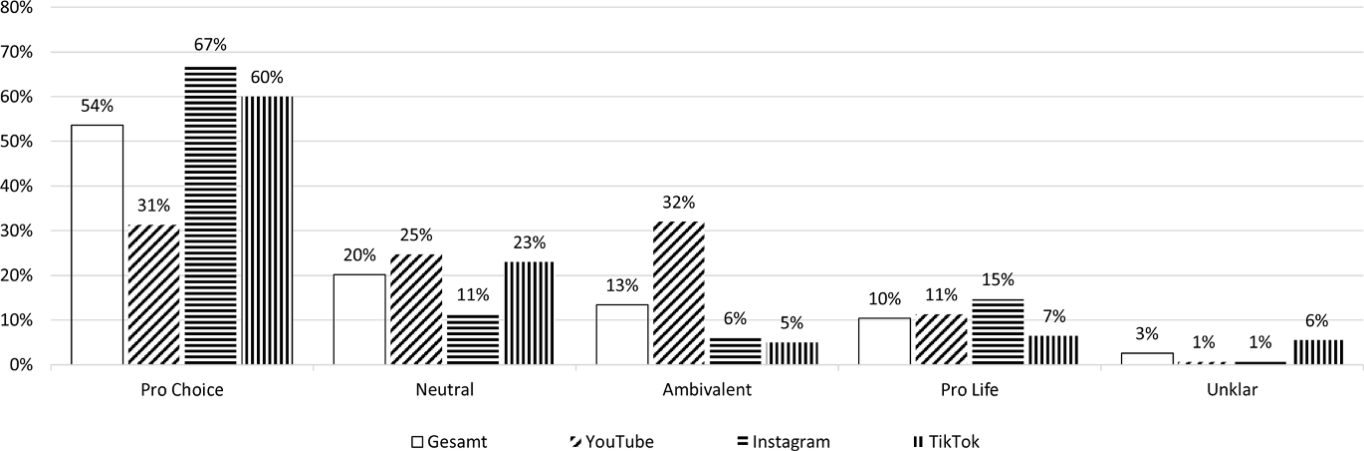
Politische Positionen in den Top-Videos zum Schwangerschaftsabbruch auf YouTube, Instagram und TikTok (in Prozent der Videos im Sample). (Prozentwerte basieren auf *N* = 500 Videos mit *n* = 150 YouTube, *n* = 150 Instagram und *n* = 200 TikTok und sind in absteigender Reihenfolge der Prozentwerte gesamt sortiert. Zweidimensionaler Chi-Quadrat-Test *χ*^*2*^(8) = 100,69, *p* < 0,001, Cramérs *V* = 0,32. Aufgrund niedriger Zellenbesetzungen wurde Fishers exakter Test mit Monte-Carlo-Approximation gerechnet)

Die große Mehrzahl der deutschsprachigen Top-Videos zum Schwangerschaftsabbruch enthielt eine politische Positionierung, nur 20 % der Videos waren diesbezüglich neutral (Abb. 1). Die dominierende Position war Pro Choice (54 %), während die Pro-Life-Position deutlich seltener vertreten war (10 %). Im Plattformvergleich gab es einige Unterschiede (zweidimensionaler Chi-Quadrat-Test: *p* < 0,001): So waren auf YouTube, wo journalistische Formate dominieren, die meisten neutralen (d. h. keine Positionierung) oder ambivalenten (d. h. Aufzeigen sowohl der Pro-Life- als auch der Pro-Choice-Position) Videos zu finden. Gleichzeitig war auf Instagram, wo mehr politische Akteur*innen aktiv sind, eine stärkere Polarisierung zu beobachten: Hier gab es den vergleichsweise höchsten Anteil von SAB-Videos mit Pro-Choice-Positionen (67 %) und mit Pro-Life-Positionen (15 %).

Neben politischen Fragen der rechtlichen Regulierung, thematisieren die deutschsprachigen Top-Videos zum Schwangerschaftsabbruch diverse gesundheitliche Aspekte, am häufigsten medizinische Versorgung und das Erleben auf psychischer und physischer Ebene (Tab. 3). Ein Beispiel ist das YouTube-Video „Abtreibung – wie funktioniert ein Schwangerschaftsabbruch mit Tabletten?“ von pro familia, in dem eine Gynäkologin den medikamentösen Abbruch hinsichtlich medizinischer Details, Erleben und Versorgung erklärt.^6^ Im Plattformvergleich wird deutlich, dass YouTube-Videos in der Regel mehrere der 8 zentralen inhaltlichen Aspekte behandeln, während sich die deutlich kürzeren Instagram- und TikTok-Videos jeweils auf wenige ausgewählte Aspekte beschränken.

**Tab. 3:** Aspekte des Schwangerschaftsabbruchs in den Top-Videos zum Schwangerschaftsabbruch auf YouTube, Instagram und TikTok (in Prozent der Videos im Sample)

Aspekte des Schwangerschaftsabbruchs	Gesamt	YouTube	Instagram	TikTok
	%	%	%	%
Rechtliche Aspekte	56,8	70,7	56,7	46,5
Versorgungsbezogene Aspekte	50,4	69,3	46,0	39,5
Erlebensbezogene Aspekte	43,4	74,7	26,0	33,0
Soziale und gesellschaftliche Aspekte	39,8	62,7	34,0	27,0
Ethische Aspekte	31,0	53,3	28,7	16,0
Medizinische Aspekte	17,2	36,0	9,3	9,0
Ökonomische Aspekte	13,0	27,3	8,0	6,0
Wissensbezogene Aspekte	12,2	32,0	5,3	2,5

*N* = 500 Videos mit *n* = 150 YouTube, *n* = 150 Instagram und *n* = 200 TikTok. Sortiert in absteigender Reihenfolge der Prozentwerte der Gesamtspalte. Da ein Video mehrere Aspekte thematisieren kann, überschreiten die Spaltensummen 100 %. Zweidimensionaler Chi-Quadrat-Test zwischen Plattform und Anzahl der thematisierten Aspekte des Schwangerschaftsabbruchs (0, 1, 2 oder 3+ Aspekte) *χ*^*2*^(6) = 107,45, *p* < 0,001, Cramérs *V* = 0,33

Hinsichtlich der Art der präsentierten Informationen in den SAB-Videos zeigte sich grob eine Dreiteilung zwischen reinem Faktenwissen (39 %), reinem Erfahrungswissen (32 %) und einer Kombination aus beidem (30 %).

### Qualität von SAB-Videos in sozialen Medien (F3)

**Abb. 2:**
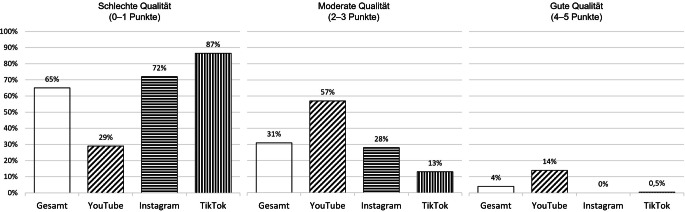
Qualität der Top-Videos zum Schwangerschaftsabbruch auf YouTube, Instagram und TikTok (gemessen mit dem mDISCERN-Index). (Prozentwerte zur Informationsqualität basieren auf *N* = 500 Videos mit *n* = 150 YouTube, *n* = 150 Instagram und *n* = 200 TikTok, gemessen mit dem mDISCERN-Index. Der mDISCERN-Gesamtwert ergibt sich aus der Summenpunktzahl der 5 Bewertungsfragen (0–1 = schlechte, 2–3 = moderate und 4–5 = gute Qualität). Sortiert in absteigender Reihenfolge der Prozentwerte gesamt. Zweidimensionaler Chi-Quadrat-Test (Informationsqualität × Plattform), *χ*^*2*^(4) = 143,53, *p* < 0,001, Cramérs *V* = 0,38)

Gemessen mit dem mDISCERN-Index hatten die Top-Videos zum Schwangerschaftsabbruch mehrheitlich (65 %) eine schlechte Informationsqualität (Abb. 2). Dabei zeigten sich deutliche Differenzen zwischen den Plattformen (zweidimensionaler Chi-Quadrat-Test: *p* < 0,001): YouTube übertraf in der Inhaltsqualität Instagram und TikTok, da YouTube-Videos gemäß den Kriterien des mDISCERN-Index häufiger auf zuverlässige Quellen verweisen, weiterführende Informationen angeben und eine ausgewogene Darstellung des Schwangerschaftsabbruchs anbieten. Beispiele für SAB-Videos auf YouTube mit hoher Informationsqualität (mDISCERN-Wert = 5) sind die gut recherchierten und mit vielen Quellen versehenen Videos „Schwangerschaftsabbruch: Was Ärzte nicht sagen dürfen“ von „Quarks“^7^ (Anbietertyp: Medienprofi) und „Schwangerschaftsabbruch | Abtreibung“ von „Gynäko.Logisch“^8^ (Anbietertyp: Gesundheitsprofi). Im Vergleich der Anbietertypen schneiden Medien- und Gesundheitsprofis mit ihren stärker auf Faktenwissen basierenden Videos beim mDISCERN-Index besser ab als Gesundheitslaien, die häufiger Erfahrungswissen teilen (Zusatzabbildung Z2 zur Qualität der Videos gemäß Anbietertypen ist dem OSF-Projekt zu entnehmen).

Dass TikTok und Instagram im mDISCERN-Index systematisch schlechter abschneiden, hängt dabei auch mit den Plattformstrukturen zusammen: Sie erlauben – im Unterschied zu YouTube – keine ausführlichen Videobeschreibungen mit Quellenangaben und weiterführenden Informationen. Ein schlechtes Abschneiden beim mDISCERN-Index ist zudem nicht gleichbedeutend mit einer hohen Fehlerrate. Wir haben das Vorkommen von 10 verschiedenen Abtreibungsmythen codiert und konnten nur insgesamt 9 Erwähnungen eines Mythos in 342 Videos mit Faktenwissen zählen (Zusatztabelle Z3 mit der Verbreitung der Mythen in den Videos ist dem OSF-Projekt zu entnehmen).

### Publikumsreaktionen auf SAB-Videos in sozialen Medien (F4)

**Abb. 3:**
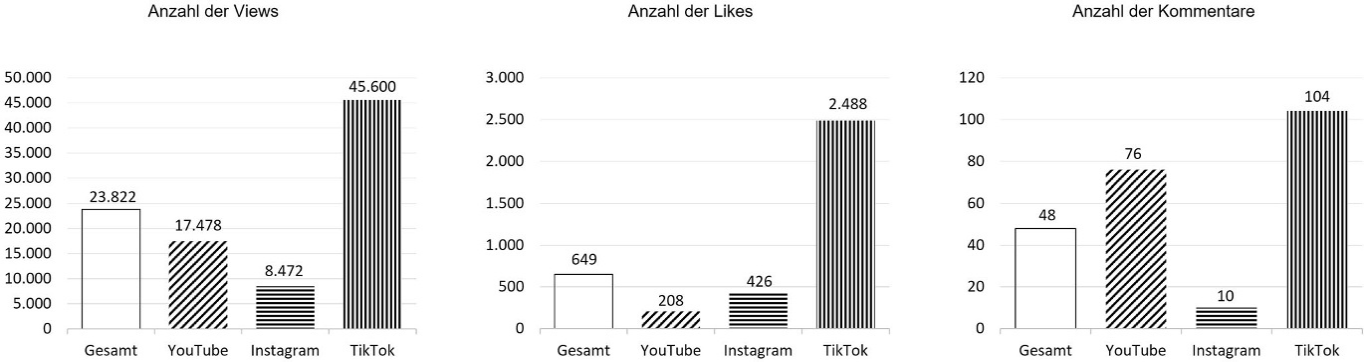
Publikationsreaktionen auf die Top-Videos zum Schwangerschaftsabbruch auf YouTube, Instagram und TikTok (Medianwerte; auf Basis von insgesamt *N* = 500 Videos). (Dargestellt werden die Medianwerte der Social-Media-Metriken von insgesamt *N* = 500 Videos zum Schwangerschaftsabbruch mit *n* = 150 YouTube, *n* = 150 Instagram und *n* = 200 TikTok. Median der *Views* pro Video; Median der *Likes* pro Video, Median der *Kommentare* pro Video)

Die untersuchten deutschsprachigen Top-Videos zum Schwangerschaftsabbruch zeigen bedeutsame Reichweiten mit einem Medianwert von 23.822 Views pro Video (Abb. 3). So hat die oben genannte Reportage „Abtreibung: Das kommt auf mich zu!“ aus dem Jahr 2017 knapp 2,5 Mio. Views und fast 20.000 Kommentare gesammelt – dabei ist sie als Top-Video weiterhin relevant, wird auch heute noch geschaut und kommentiert. Deutlich größere Interaktionsraten als YouTube und Instagram erreicht jedoch TikTok: Hier erlangten die untersuchten Top-SAB-Videos mit Abstand die meisten Views, Likes und Kommentare.

Betrachtet man die Inhalte der rund 50 Kommentare pro Video (Abb. 3), so zeigt die Analyse unserer Stichprobe der maximal 20 meistgelikten themenbezogenen Kommentare folgende Ergebnisse:

- *Politische Positionen des Publikums*: Die meisten Publikumskommentare (90 %) enthielten eine politische Positionierung zum Schwangerschaftsabbruch, wobei Pro-Choice-Positionen (51 %) verbreiteter waren als Pro-Life-Positionen (23 %; Zusatzabbildung Z4 mit den Kommentarpositionen im Plattformvergleich ist dem OSF-Projekt zu entnehmen).
- *Persönliche Erfahrungen des Publikums*: Ein Teil der Kommentare (12 %) enthielt persönliche Erfahrungsberichte, die sich etwa um die Entscheidungsfindung vor dem Abbruch und Schmerzen durch den Abbruch drehen oder um eine rückblickende Reflexion: „Ich habe es bis heute nicht bereut, aber ich denke ab und zu daran zurück und würde gern wissen, ob es ein Mädchen oder ein Junge geworden wäre.“
- *Zusatzinformationen durch das Publikum*: Zuweilen ergänzt das Publikum in den Kommentaren die Inhalte des zugehörigen Videos, indem es selbst recherchierte Fakten und Quellen angibt, etwa amtliche Statistiken zur Verbreitung von Abbrüchen oder Informationen zur Kostenübernahme durch Krankenkassen (8 %).
- *Nachfragen des Publikums*: Nicht zuletzt nutzt das Publikum die Kommentarspalten zuweilen auch (6 %), um Fragen an die Videoanbieter*innen oder andere Zuschauer*innen zu richten, etwa zu medizinischen, ökonomischen oder ethischen Aspekten.

## Diskussion

### Interpretation der Befunde

Die vorliegende Studie zeigt, dass die populärsten deutschsprachigen Videos zum Schwangerschaftsabbruch auf den führenden Social-Media-Plattformen YouTube, Instagram und TikTok mehrheitlich von Medienprofis und von Gesundheitslaien stammen, während Gesundheitsprofis nur einen kleinen Anteil stellen (F1). Diese Anbieterstruktur ähnelt Ergebnissen aus Analysen zu englischsprachigen SAB-Videos [34, 36] sowie zu deutschsprachigen Social-Media-Beiträgen mit anderen reproduktiven Gesundheitsthemen (z. B. Verhütung [44]).

Inhaltlich geht es in den Videos oft um eine politische Positionierung, die mehrheitlich im Sinne der Pro-Choice-Position ausfällt, sowie um verschiedene gesundheitliche Aspekte, etwa Fragen der medizinischen Versorgung (F2). Dieser Befund entspricht Erkenntnissen über Inhalte englischsprachiger SAB-Videos [20, 34].

Legt man an die SAB-Videos Kriterien für hochwertige Gesundheitsinformationen an, wie sie der mDISCERN-Index misst, dann zeigen sich starke Defizite, besonders bei den Kurzvideos auf Instagram und TikTok (F3). Das entspricht Befunden aus der internationalen Forschung [18] und liegt vor allem daran, dass diese Videos aufgrund ihrer Kürze nur einzelne Aspekte des Themas anschneiden und zudem keine weiterführenden Informationen und Quellenangaben liefern. Dezidierte Falschinformationen im Sinne von Abtreibungsmythen traten jedoch nur selten auf. Zudem ist zu beachten, dass neben evidenzbasiertem Faktenwissen auch das Erfahrungswissen anderer Frauen für Betroffene hilfreich sein kann – dies wird jedoch bislang in der Qualitätsmessung nicht berücksichtigt.

Betrachtet man die Publikumsreaktionen auf die populären deutschsprachigen SAB-Videos, so zeigt sich, dass vor allem über TikTok das Thema sehr große Reichweiten erzielt und die Kommentarspalten ein Forum bieten für politische Positionierung und persönlichen Erfahrungsaustausch des Publikums (F4).

### Limitationen

Soziale Medien und ihre Inhalte sind definitionsgemäß sehr dynamisch. Die vorliegende Studie ist dementsprechend eine Momentaufnahme. Ein regelmäßiges Monitoring wäre notwendig, um den aktuellen Stand der Social-Media-Kommunikation im Zeitverlauf mitzuverfolgen. Nachdem hier ein relativ großes Sample an reichweitenstarken deutschsprachigen Videos und zugehörigen Kommentaren von 3 verschiedenen Plattformen untersucht wurde, könnten in zukünftigen Studien reichweitenärmere Inhalte sowie SAB-Informationen von weiteren sozialen Medien (z. B. Facebook, X/Twitter, Twitch) sowie auch von KI-Tools (z. B. Gemini, ChatGPT, Claude, DeepSeek) einbezogen werden. Messinstrumente zur Qualität von Social-Media-Gesundheitsinformationen gilt es weiterzuentwickeln. Denn der etablierte mDISCERN-Index ist (a) einseitig auf Faktenwissen ausgerichtet, vernachlässigt somit Qualität und Nutzen von geteiltem Erfahrungswissen und (b) berücksichtigt keine Plattformbesonderheiten (z. B. die vorhandene oder nicht vorhandene Möglichkeit, einem Video eine Beschreibung mit Quellenangaben beizufügen).

### Fazit

Die vorliegende Inhalts- und Qualitätsanalyse beschreibt die SAB-Kommunikation auf YouTube, Instagram und TikTok. Dabei bietet YouTube im Plattformenvergleich die gehaltvollsten Beiträge, meist Pressereportagen, und auch die längsten Kommentare – hier werden am ehesten verschiedene Aspekte des Themas angesprochen. Auf Instagram ist die SAB-Darstellung am stärksten durch politischen Aktivismus geprägt. Auf TikTok bekommt ein sehr junges Publikum knappe Clips vom Algorithmus zugespielt, was nicht selten zu Nachfragen führt. Für die professionelle Sexualaufklärung ergeben sich die beiden Anforderungen, (a) mit qualitätsvollem eigenen Content präsent zu sein sowie (b) durch zeitgemäße sexualbezogene Medienbildung die Social-Media-Nutzenden zu befähigen, mit Beiträgen und Kommentaren zum Schwangerschaftsabbruch kompetent und kritisch umzugehen.

## Data Availability

Die Studie ist präregistriert und folgt dem Open-Science-Ansatz, das heißt, alle Codebücher, Datensätze, Auswertungsskripte sowie zusätzliche Ergebnistabellen und -abbildungen Z1–Z4 sind auf dem Server des Open Science Framework (OSF) öffentlich zugänglich hinterlegt: https://osf.io/9ew4k/.

## Footnotes

1 Zusätzlich ist ohne Pflichtberatung ein Schwangerschaftsabbruch legal möglich gemäß medizinischer (§ 218a Abs. 2 StGB) oder kriminologischer (§ 218a Abs. 3 StGB) Indikation.

2 Arztpraxen in Deutschland dürfen seit 2022 – also seit der Streichung des § 219a StGB („Werbung für den Abbruch der Schwangerschaft“) – auf ihren Webseiten sachlich und berufsbezogen über Schwangerschaftsabbrüche informieren.

3 Das gilt für unsere Studie insbesondere deswegen, weil die Videos von reichweitenstarken Accounts stammen, die sich ihrer Öffentlichkeitswirkung bewusst sind, und da wir Videokommentare nur anonym zitieren. Für weitere Details zur ethischen Verwendung von Social-Media-Material siehe z. B. [40].

4 https://www.youtube.com/watch?v=7odoqTw4aVg (letzter Zugriff: 05.08.2025).

5 https://www.tiktok.com/@evil_suki/video/7158463608605756678 (letzter Zugriff: 05.08.2025).

6 https://www.youtube.com/watch?v=Lo6QGQkQqcQ&t (letzter Zugriff: 05.08.2025).

7 https://www.youtube.com/watch?v=-sg83BalRrs (letzter Zugriff: 05.08.2025).

8 https://www.youtube.com/watch?v=qZg4WgelJ1A (letzter Zugriff: 05.08.2025).
